# Hypoactivity and neurochemical alterations in the basal ganglia of female Sprague-Dawley rats after repeated exposure to atrazine

**DOI:** 10.3389/ftox.2024.1416708

**Published:** 2024-08-05

**Authors:** Triana Acevedo-Huergo, Jonathan Sánchez-Yépez, María Soledad Mendoza-Trejo, Isela Hernández-Plata, Magda Giordano, Verónica Mireya Rodríguez

**Affiliations:** Departamento de Neurobiología Conductual y Cognitiva, Instituto de Neurobiología, Universidad Nacional Autónoma de México, Querétaro, Mexico

**Keywords:** GABA, glutamate, dopamine, serotonin, locomotor activity, herbicides, toxicity

## Abstract

The herbicide atrazine (ATR) has been one of the most widely used herbicides worldwide. However, due to its indiscriminate use, it has been considered an environmental contaminant. Several studies have classified ATR as an endocrine disruptor, and it has been found to have neurotoxic effects on behavior, along with alterations in the dopaminergic, GABAergic, and glutamatergic systems in the basal ganglia of male rodents. These findings suggest that these neurotransmitter systems are targets of this herbicide. However, there are no studies evaluating the neurotoxicity of ATR in female rodents. Our study aimed to assess the effects of repeated IP injections of 100 mg ATR/kg or a vehicle every other day for 2 weeks (six injections) on the locomotor activity, content of monoamines, GABA, glutamate, and glutamine in the striatum, nucleus accumbens, ventral midbrain, and prefrontal cortex, and tyrosine hydroxylase (TH) protein levels in striatum and nucleus accumbens of female rats. Repeated 100 mg ATR/kg injections immediately decreased all the locomotor activity parameters evaluated, and such hypoactivity persisted for at least 48 h after the last ATR administration. The ATR administration increased dopamine and DOPAC content in the nucleus accumbens and the dopamine and DOPAC and serotonin and 5-HIAA content in the ventral midbrain. In contrast, the TH protein levels in the striatum and nucleus accumbens were similar between groups. Meanwhile, GABA, glutamine, and glutamate levels remained unaltered in all brain regions evaluated. The observed behavioral alterations could be associated with the monoamine changes presented by the rats. These data reveal that the nucleus accumbens and ventral midbrain are susceptible to repeated ATR exposure in female rats.

## Introduction

Atrazine (6-chloro-N-ethyl-N-isopropyl-1, 3, 5-triazine-2, 4-diamine; ATR) is a broad-spectrum herbicide used to control or eliminate the growth of weeds in various crops such as corn, pineapple, sugar cane, among others (Shaner et al., 2011). ATR’s global commercial introduction was in 1958. Furthermore, the continuous and widespread application of ATR is considered an environmental pollutant (Garrido et al., 2000; Barbash et al., 2001; Gilliom et al., 2006; Meffe and de Bustamante, 2014).

Studies in different animal models have shown that ATR is an endocrine disruptor (defined as an environmental pollutant capable of acting as agonist/antagonist or modulator of the metabolism of neurohormones, neuropeptides, or neurotransmitters, which alter behavioral, hormonal, or physiological processes that affect the capacity to develop, grow, or reproduce, among other deleterious effects in exposed organisms (Waye and Trudeau, 2011) with ontogenetic, reproductive, and immunological effects (Cooper et al., 2000; Hayes et al., 2002; 2015; Kass et al., 2020). In addition, it has been demonstrated that ATR can cross the blood-brain barrier through still-unknow mechanisms (Ross et al., 2008). Several treatment paradigms, such as repeated and chronic exposure to ATR, cause alterations in the nigrostriatal dopaminergic system, the GABAergic and glutamatergic systems in the basal ganglia, and some other brain structures of male rats. Meanwhile, male rats that were repeatedly exposed to 100 mg ATR/kg (six intraperitoneal injections) over 2 weeks showed hypoactivity after each injection, which lasted up to 5 days after the last injection, decreased the striatal DA levels and the TH and presynaptic dopamine transporter mRNA levels but increased the vesicular monoamine transporter-2 mRNA levels in the ventral midbrain (Rodríguez et al., 2013). Under the same exposure paradigm, six IP injections of 100 mg ATR/kg decreased the specific binding of the [3H]-SCH23390 antagonist to dopamine D1 receptors in the striatum of male rats (Márquez-Ramos et al., 2017).

On the other hand, chronic daily exposure to 10 mg ATR/kg for 1 year reduces the striatal levels of DA, causes hyperactivity, and decreases the counts of TH + cells on the SNpc of male rats (Bardullas et al., 2011; 2013) and recent studies also using male rats reported that chronic exposure to 1 or 10 mg ATR/kg during 14 months produces hyperactivity and anxiety, which were associated with increased extracellular levels of striatal glutamate, alterations in GABA, glutamate, and glutamine levels, and increased expression of genes associated to the metabolism of GABA and glutamate in the striatum, nucleus accumbens, ventral midbrain, amygdala, and the prefrontal cortex (Chávez-Pichardo et al., 2020; Reyes-Bravo et al., 2022). These reports suggest that the alterations seen in the behavior of male rodents may be associated with alterations not only in the dopaminergic system but also in the glutamatergic and GABAergic systems.

It is essential to mention that in the reports cited above, male rodents were mainly used as experimental models. Although using males is essential, it was not until 2014 that the NIH established policies to include male and female animals in experimental designs due to a lack of pre-clinical studies using both sexes. There is growing evidence of the sex-dependent effects of pesticides in different animal models, including ATR (Torres-Rojas and Jones, 2018). In this respect, it has been reported that developmental exposure from gestational day 10 through postnatal day 23–35 mg ATR/kg to pregnant Sprague-Dawley dams caused suppression of humoral function in males but not in females offsprings (Rooney et al., 2003). While in female and male Fisher rats, the administration of 120 mg of ATR/kg orally for 7 days caused loss of body weight in both sexes, in addition to a significant increase in the relative weights of the pituitary and prostate in males and deregulation in the estrous cycle of females (Šimić et al., 1994). It is worth mentioning that recent work by our group demonstrated that female rats chronically treated with 1 or 10 mg ATR/kg for 14 months do not present alterations in the tissue levels of dopamine or DOPAC in the striatum, nucleus accumbens, prefrontal cortex, or ventral midbrain (Sánchez-Yépez et al., 2024). Since it has been widely reported that ATR affects the dopaminergic, glutamatergic, and GABAergic systems in male rats, the present work aimed to evaluate whether repeated exposure to ATR causes toxic effects on locomotor activity and in dopaminergic, serotonergic, glutamatergic, and GABAergic systems of the female rat.

## Methods

### Chemicals

ATR was obtained from ChemService (98% purity, West Chester, PA, USA). Reagents for HPLC-ED were obtained from Sigma-Aldrich (St. Louis, MO, United States), and reagents for Western blot were obtained from BioRad (Hercules, CA, USA) unless otherwise stated.

### Animals

Thirty-five, two months-old female Sprague-Dawley rats weighing 165–180 g were acquired from the vivarium of the Instituto de Neurobiologia-UNAM. They were kept under a 12 h inverted dark/light cycle (lights on at 8:00 p.m.) with access to food and water *ad libitum*.

### Ethics

All experiments were approved by the local Committee of Bioethics and carried out outfitting to the Official Mexican Standard NOM- 062-ZOO-1999 titled “Especificaciones técnicas para la producción, cuidado y uso de los animales de laboratorio,” which agrees with the guidelines of the Institutional Animal Care and Use Committee Guidebook (NIH Publication 80–23, Bethesda, MD, United States, 1996).

### Experimental design

Rats were allowed to habituate to the laboratory for 1 week and then they were randomly distributed into four groups. Two groups of rats (one vehicle and one ATR; n = 8–10) were used to determine monoamines and protein expression of tyrosine hydroxylase (experiment 1). The other two groups (one vehicle and one ATR) were used to record locomotor activity (n = seven to eight per group). Rats from locomotor activity groups were randomly selected into batches (n = 5 per group) to determine GABA, glutamate, and glutamine (experiment 2).

All rats received six IP injections of 1% methylcellulose (VEH) or 100 mg ATR/kg BW over 2 weeks (three injections per week with 48 h intervals). This dose has been previously used by our group in similar protocols performed in male Sprague-Dawley rats (Rodríguez et al., 2013; Marquez-Ramos et al., 2017). ATR administration was under “Neurotoxicity Screening Battery Health Effects Test Guidelines”, OPPTS, 870.6200 [A-96–16-II-I-3], available at the website (https://www.regulations.gov/document/EPA-HQ-OAR-2003-0065-0783).

Rats received an IP saline injection 2 days after the last ATR or VEH administration. For the rats of experiment 2, the locomotor activity was evaluated 15 min before the IP administration of VEH or 100 mg ATR/kg and 2 hours after the injection of VEH, ATR, or saline.

For experiment 1, 2 hours after the last administration of saline, rats were sacrificed by decapitation, from one hemisphere was extracted, the striatum, nucleus accumbens, ventral midbrain, and prefrontal cortex were dissected for monoamines determination of DA and serotonin (5-HT) and their metabolites, while for the other hemisphere striatum and nucleus accumbens were collected for tyrosine hydroxylase protein expression, all brain tissues were frozen at −80°C until processing. Meanwhile, for experiment 2, at the end of locomotor activity recording, rats were sacrificed for the determination of GABA, glutamate, and glutamine in the striatum, nucleus accumbens, ventral midbrain, and prefrontal cortex.

### Locomotor activity

Locomotor activity was assessed, as reported earlier by our group (Rodríguez et al., 2013; Marquez-Ramos et al., 2017). Briefly, rats were individually placed in an automated locomotor activity chamber equipped with horizontal and vertical infrared beams (Accuscan Instruments Inc., Columbus, OH, USA). Before each injection, rats were allowed to explore the locomotor activity chamber for 15 min, after which they were injected with 100 mg ATR/kg BW, VEH, or saline, and their activity was recorded for 2 h. The first 15 min were defined as an exploration period and analyzed separately from the 2-h recording. The locomotor activity parameters evaluated were the total distance, defined as the distance in cm traveled by the rat during a given period; horizontal activity, defined as total number of beam interruptions that occurred in the horizontal sensor during a given period; vertical activity, defined as the total number of beam interruptions that occurred in the vertical sensor during a given period; and stereotypy counts (stereotypy), defined as the number of beam breaks that occurred during recurring movements such as grooming, head bobbing, among others during a given period.

### Monoamines determination

The striatum, nucleus accumbens, ventral midbrain, or prefrontal cortex was sonicated in 500 uL of 0.1 N perchloric acid and centrifuged at 10,000 rpm at 4°C for 20 min. The supernatant was collected and immediately frozen at −80°C until monoamine determination. The pellet was air-dried for 3 days, and later, it was digested in 500 uL of 0.5 M NaOH and used for protein determination with the Lowry DC technique (BioRad, Hercules, CA, USA). 35 uL of supernatant was injected by an autosampler (PerkinElmer, Waltham, MA, USA) into a series 200 PerkinElmer pump, coupled to a chromatographic column packed with Alltima 3 um C18 (100 × 4.6 mm; Hichrome, Leicestershire, United Kingdom). An electrochemical detector LC-4C (Bioanalytical system, West Lafayette, IN, USA) set at 0.850 V relative to a silver/silver chloride electrode and a sensitivity of 10 nA was coupled to the chromatographic system. The mobile phase was an aqueous, isocratic 0.1 M monobasic phosphate solution containing 0.5 mM sodium octyl sulfate, 0.03 mM EDTA, and 12%–14% (v/v) methanol. The elution was performed at room temperature for 30 min at a 1 mL/min flow rate. External standards were used to construct calibration curves for DA, 5-HT, and their metabolites. The results were analyzed with TotalChrom Navigator version 6.3.1.0504 (PerkinElmer), and concentrations were expressed in ng per mg of protein.

### GABA, glutamate, and glutamine determination

Rats received an intraperitoneal injection of 1.2 mmol/kg of 3-mercaptopropionic acid, an inhibitor of glutamate decarboxylase (Sprince et al., 1970; Löscher, 1979); 2 minutes later, they were decapitated. The brain was collected, and the striatum, nucleus accumbens, prefrontal cortex, and ventral midbrain were dissected. Briefly, each tissue was sonicated in 1 mL of 85% methanol and centrifuged at 10,000 rpm at 4°C for 20 min. The supernatant was collected and immediately stored at −80°C until amino acids determination. The pellet was air-dried for 3 days, and later, it was digested in 1,000 uL of 0.5 M NaOH and used for protein determination with the Lowry DC technique (BioRad). 30 uL of supernatant was pre-derivatized with 13 uL of O-phthalaldehyde (OPA) solution (5 mM OPA, 1.77 mM beta-mercaptoethanol, 0.25% methanol, 0.1 M borate buffer, pH = 9.3) and 40 uL were injected by an autosampler (PerkinElmer) into a series 200 PerkinElmer pump, coupled to an adsorbosphere OPA HS 5um column (Grace, Deerfield, IL, USA) with an electrochemical detector LC-4C (Bioanalytical system) set at 0.400 V relative to a silver/silver chloride electrode, and sensitivity of 10 nA. The mobile phase A was 50 mM sodium phosphate buffer and 15% methanol, while phase B was 100% methanol. The elution was performed at room temperature, employing a linear gradient from 0% to 30% solution B (15%–40.5% methanol) over 70 min at a 1.2 mL/min flow rate (Erdö et al., 1984; Castillo et al., 2008). External standards were used to construct calibration curves for GABA, glutamate, and glutamine. The results were analyzed with TotalChrom Navigator version 6.3.1.0504 (PerkinElmer), and concentrations were expressed in ng per mg of protein.

### Tyrosine hydroxylase levels

Tyrosine hydroxylase levels were assessed by Western blot technique according to Hernández-Plata et al. (2015). Briefly, a portion of nucleus accumbens or striatum was sonicated individually in a lysis buffer (Pan et al., 2006). Each tissue sample (5 µg of protein) was separated by electrophoresis on 12% SDS-PAGE and transferred to PVDF membranes using a semidry blotting apparatus (BioRad). Membranes were blocked, and later they were incubated with anti-TH antibody (1:5,000, Millipore, Cat# AB152, RRID: AB_390204, Temecula, CA, USA) for 12 h at 4°C, followed by anti-GAPDH antibody (Glyceraldehyde-3-phosphate dehydrogenase, 1:5,000, Cell Signaling Technology, Cat# 5174, Danvers, MA, USA) during 12 h at 4°C. Later, we added the anti-rabbit secondary antibody conjugated to horseradish peroxidase for 5 h (1:5,000, Cell Signaling Technology Cat# 7074s). The detection was performed with an ECL-Plus kit (GE, Buckinghamshire, United Kingdom) and high-performance chemiluminescence film (GE). We used a fixer and developer (Carestream Dental, Atlanta, GA, USA) to detect the signal in the films. The density of each protein band was quantified using ImageJ ver. 5.0 software (NIH, Baltimore, MD, USA). Six or seven samples were analyzed per treatment of both striatum and nucleus accumbens, respectively.

### Statistical analysis

To analyze body weight and spontaneous locomotor activity, we performed a two-way repeated measures analysis of variance (RMANOVA; treatment by ATR injection) followed by a *post hoc* Student’s t-test. In contrast, TH levels and tissue levels of monoamines, GABA, glutamate, and glutamine tissue levels were evaluated with Mann-Whitney’s U tests. The Shapiro-Wilk test was used to evaluate normality. The significance level p < 0.05 was considered in all cases.

## Results

### Body weight and general appearance

**FIGURE 1:**
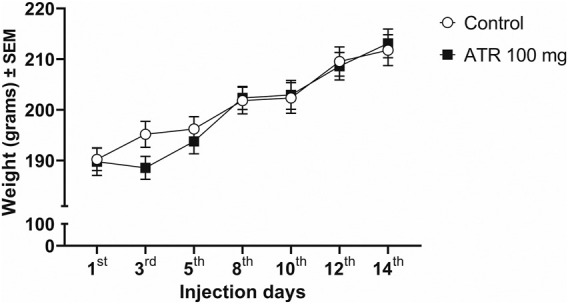
Growth rate (in grams) of the female rats for the duration of the experiment (n = 17–18 per group).

The repeated exposure to 100 mg ATR/kg does not modify the general appearance of rats, nor causes alterations in the body weight [F (1, 33) = 0.1251, p = 0.7258], nor the interaction (treatment x ATR injection days) [F (6, 198) = 1.773, p = 0.1063]. However, there were effects of ATR injection days [F (6,198) = 70.28, p < 0.0001], which represents the average growth of rodents (Figure 1).

### Repeated ATR exposure produces hypoactivity

#### ATR exposure decreases exploratory activity

**FIGURE 2:**
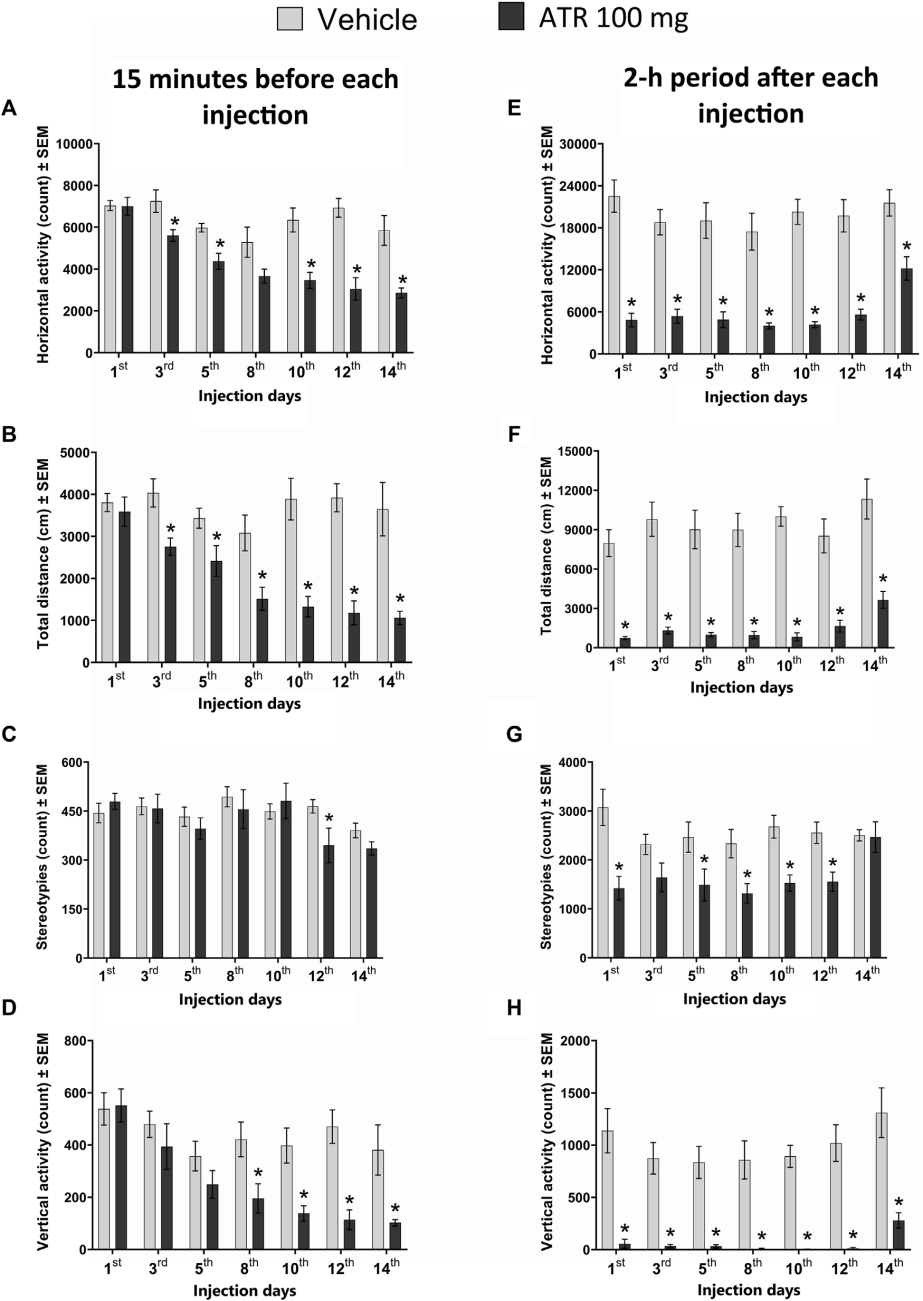
Locomotor activity recorded 15 min before **(A–D)** and during the 2-h period **(E–H)** immediately following the administration of 100 mg ATR/kg (six doses over 14 days), vehicle or saline (2 days after the last injection of ATR). *Different from the vehicle group (VEH), p < 0.05 (n = seven to eight per group).

Our statistical analysis revealed a decrease in the total distance traveled 48 h after the first ATR administration and the subsequent injections. The repeated measures ANOVA showed significant ATR treatment effect [F (1, 15) = 40.90, p < 0.0001], injection day effects [F (6, 90) = 5.348, p = 0.028], and interaction effect (ATR treatment x injection day) [F (6, 90) = 4.264, p = 0.0008]. The *post hoc* analysis further confirmed significant decreases due to ATR treatment for the injections 2–7 [t’s (15) = 2.394–6.148, p < 0.05] (Figure 2A).

For the horizontal activity, the repeated measures ANOVA indicated significant ATR treatment effect [F (1, 15) = 36.3, p < 0.0001], injection day effects [F (6, 90) = 10.8, p < 0.0001], and interaction effect (ATR treatment x injection day) [F (6, 90) = 4.23, p = 0.0009]. The *post hoc* analysis revealed decreases in horizontal activity due to ATR treatment for injection 2 [t = 2.614, p = 0.0195], injection 3 [t = 3.824, p = 0.0017], injection 5 [t = 4.072, p = 0.001], injection 6 [t = 5.594, p < 0.0001], and injection 7 [t = 3.786, p = 0.0018] (Figure 2B).

For the number of stereotypies, there was no ATR treatment effect [F (1, 15) = 0.7729, p = 0.3932], neither interaction effect (ATR treatment x injection day) [F (6, 90) = 1.622, p = 0.1501]. However, a significant effect for injection day was found [F (6, 90) = 3.873, p = 0.0017] (Figure 2C).

Regarding the vertical activity, there was a significant ATR treatment effect [F (1, 15) = 12.8, p = 0.0027], injection day effects [F (6, 90) = 8.134, p < 0.0001], and interaction effect (ATR treatment x injection day) [F (6, 90) = 2.893, p = 0.0126]. The *post hoc* analysis revealed decreases in the vertical activity due to ATR treatment for injection 4 [t = 2.564, p = 0.0216], injection 5 [t = 3.392, p = 0.004], injection 6 [t = 4.652, p = 0.0003], and injection 7 (saline solution) [t = 2.705, p = 0.0163] (Figure 2D).

#### Acute ATR exposure decreases locomotor activity recorded for 2 hours

The statistical analysis unveiled significant effects of ATR treatment [F (1, 15) = 115.3, p < 0.0001] and the injection day effects [F (6, 90) = 2.496, p = 0.028] on the total distance. Notably, there was no significant interaction (ATR treatment x injection day) effect [F (6, 90) = 0.374, p = 0.8937]. The *post hoc* analysis further showed significant decreases between the vehicle and the ATR group after all the injections [t’s (15) = (4.438–10.96), p < 0.05] (Figure 2E).

For the horizontal activity, significant ATR treatment [F (1, 15) = 94.27, p < 0.0001] and the injection day effects were found [F (6, 90) = 3.095, p = 0.0312]. However, no significant interaction (ATR treatment x injection day) effect was found [F (6, 90) = 1.342, p = 0.2469]. The *post hoc* analysis showed significant reductions in activity after ATR treatment compared to the vehicle after every injection [t’s (15) = (3.674–8.291), p < 0.05] (Figure 2F).

Our study also revealed a significant decrease in the number of stereotypies following ATR treatment [F (1, 15) = 19.55, p = 0.0005]. The injection day effect [F (6, 90) = 1.756, p = 0.01563] and significant interaction (ATR treatment x injection day) effect [F (6, 90) = 2.299, p = 0.0413] further support this finding. The *post hoc* analysis showed significant decreases for all ATR injections [t’s (15) = (1.916–3.922), p < 0.05], but there were no effects after injection 7 (Figure 2G).

For vertical activity, there were significant effects for ATR treatment [F (1, 15) = 85.86, p < 0.0001] and the injection day [F (6, 90) = 2.24, p = 0.0464]. No interaction effect (ATR treatment x injection day) was found [F (6, 90) = 0.3772, p = 0.8919]. The *post hoc* tests showed significant decreases in this parameter in all the injections of the group treated with ATR in comparison to the control group [t’s (15) = (3.939–7.907), p < 0.05] (Figure 2H).

### Repeated ATR exposure alters dopamine and DOPAC in the nucleus accumbens and DA and 5-HT and 5-HIAA in the ventral midbrain but not in the dorsal striatum.

In the nucleus accumbens, there were significantly increased levels of dopamine [U = (17), p = 0.0434] and its metabolite DOPAC [U = (16), p = 0.0343] in the group exposed to 100 mg ATR in comparison to the control group. No alterations were found in this brain region’s HVA, serotonin, metabolite levels, or DOPAC/DA turnover. Similarly, in the ventral midbrain, there were significantly increased tissular levels of DA [U = (12), p = 0.0117] and its metabolite DOPAC [U = (7), p = 0.0021], 5-HT [U = (4), p = 0.0005] and its metabolite 5-HIAA [U = (7), p = 0.0021] in comparison to the control group. Nevertheless, there were no alterations in the content of HVA. While in the striatum and the prefrontal cortex there were no significant effects in the tissue levels of dopamine, serotonin, or their metabolites (DOPAC, HVA, or 5-HIIA) in the group treated with 100 mg ATR compared to the control group (Table 1).

**TABLE 1:** Concentrations of dopamine, serotonin and their metabolites.

	DA	DOPAC	HVA	DA/DOPAC	5-HT	5-HIAA
*Striatum*						
Control	315.1 ± 35.84	55.68 ± 12.08	41.56 ± 6.76	0.18 ± 0.04	20.55 ± 3.25	16.71 ± 6.27
ATR 100 mg	251.9 ± 26.39	45.3 ± 12.97	29.87 ± 2.76	0.17 ± 0.04	16.45 ± 1.38	17.08 ± 5.15
*Nucleus accumbens*						
Control	121.7 ± 10.67	7.67 ± 1.18	13.58 ± 1.97	0.067 ± 0.009	N.D.	5.86 ± 2.23
ATR 100 mg	162.8 ± 14.05 *	12.76 ± 1.82 *	18.25 ± 1.80	0.078 ± 0.009	N.D.	6.36 ± 0.80
*Ventral midbrain*						
Control	5.65 ± 0.89	3.44 ± 0.51	22.61 ± 5.92	0.63 ± 0.09	15.41 ± 2.60	10.09 ± 1.74
ATR 100 mg	12.24 ± 2.03 *	22.18 ± 13.42 *	29.6 ± 9.61	1.28 ± 0.46	29.6 ± 3.018 *	27.6 ± 3.42 *
*Prefrontal cortex*						
Control	0.83 ± 0.08	N.D.	1.84 ± 0.88	N.D.	11.52 ± 1.19	3.96 ± 0.45
ATR 100 mg	0.63 ± 0.06	N.D.	1.46 ± 0.58	N.D.	8.25 ± 1.35	3.20 ± 0.43

Values are mean ± SEM (n = 8–10) as ng/mg of protein. DA, dopamine; DOPAC, dihydroxyphenylacetic acid; HVA, homovanillic acid; 5-HT, serotonin; 5-HIAA, 5-hydroxyindolacetic acid; N.D., no detected.

* and bold digits indicate significant differences with respect to the control group.

#### Tyrosine hydroxylase levels

**FIGURE 3:**
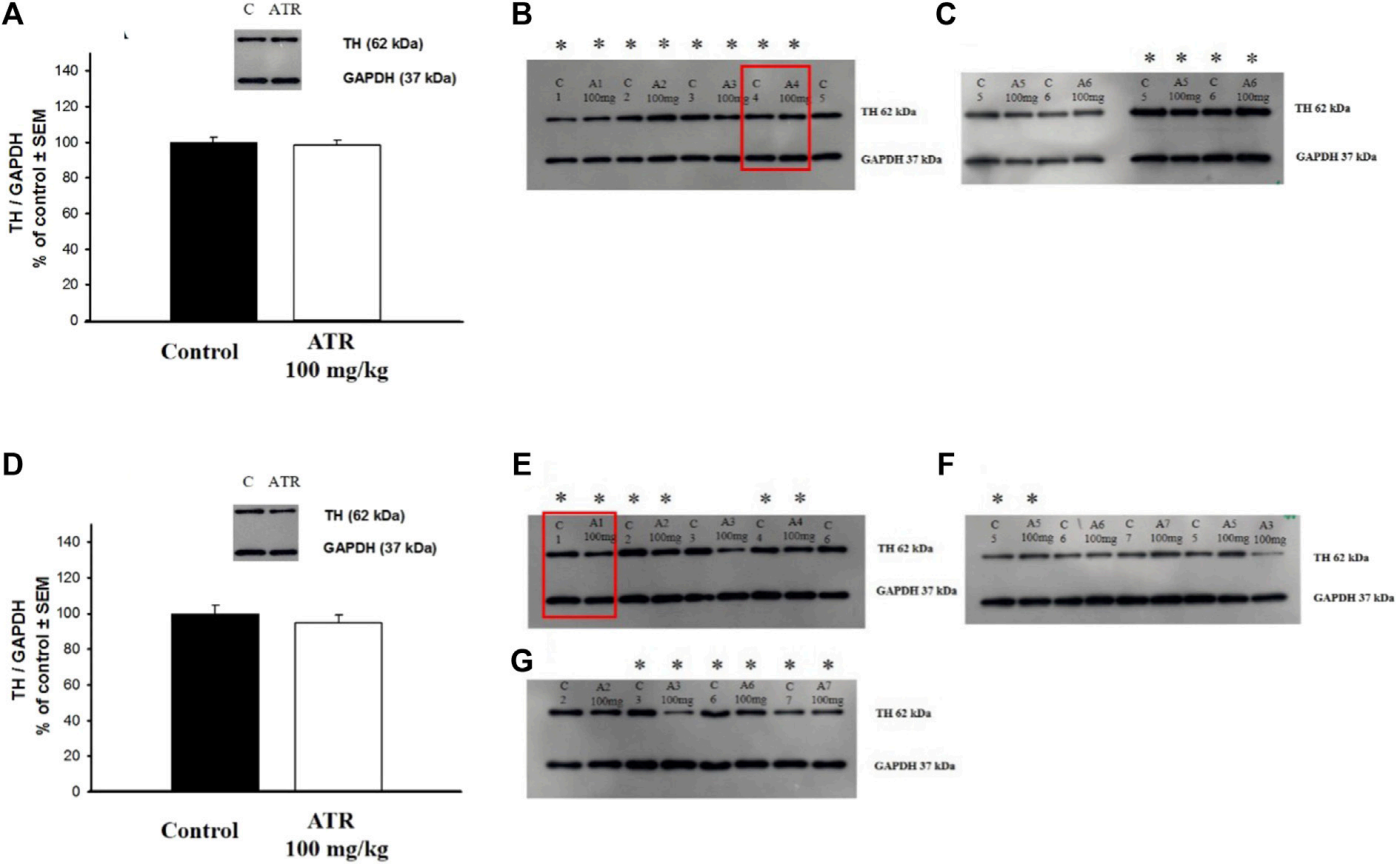
Tyrosine hydroxylase (TH) levels in the striatum **(A–C)** and the nucleus accumbens **(D–G)**. Data is expressed as a percentage of the control group. C = control, A = Atrazine. * Indicates the blots considered for the analysis. The blots used in the main figure are inside red rectangles. (n = six to seven per group).

We found that the six injections of 100 mg ATR did not affect TH levels in the striatum (Figures 3A–C) or nucleus accumbens (Figures 3D–G).

### Repeated ATR exposure does not alter GABA, glutamate, and glutamine levels in brain tissues

No significant differences due to 100 mg ATR injections were found in GABA, glutamate, or glutamine levels in the various brain structures evaluated (Table 2).

**TABLE 2:** Concentrations of glutamate, glutamine and GABA.

	Glutamate	Glutamine	GABA
*Striatum*			
Control	15.77 ± 3.66	2.89 ± 0.42	2.05 ± 0.30
ATR 100 mg	22.75 ± 5.55	3.17 ± 0.51	2.59 ± 0.28
*Nucleus accumbens*			
Control	12.08 ± 1.81	2.28 ± 0.50	5.02 ± 0.82
ATR 100 mg	11.98 ± 1.53	2.27 ± 0.34	4.37 ± 0.58
*Ventral midbrain*			
Control	10.29 ± 0.94	1.07 ± 0.093	6.30 ± 0.59
ATR 100 mg	9.65 ± 1.49	0.98 ± 0.20	6.30 ± 0.82
*Prefrontal cortex*			
Control	10.49 ± 1.46	1.43 ± 0.12	1.59 ± 0.16
ATR 100 mg	11.69 ± 1.36	1.30 ± 0.31	1.75 ± 0.13

Values are mean ± SEM (n = 5) as ng/mg of protein.

#### Oestrus cycle

The estrous cycle of female rats was evaluated immediately before euthanasia. Regardless of the treatment group, most rats were in the diestrus and proestrus (Table 3).

**TABLE 3:** Number of rats in each phase of the oestrus cycle.

Experiment 1 Monoamine levels				
	Proestrus	Estrus	Metestrus	Diestrus
Control	1	2	1	4
ATR 100 mg/kg	4	1	0	5

Experiment 2 Locomotor activity recording and GABA, glutamate and glutamine levels				
	Proestrus	Estrus	Metestrus	Diestrus
Control	2	2	1	0
ATR 100 mg/kg	1	0	1	3

## Discussion

Our investigation showed that ATR exposure affected monoaminergic systems in female rats, without altering the GABAergic or glutamatergic systems. Specifically, repeated 100 mg ATR/kg administration increased dopamine levels and its metabolite DOPAC in female rats’ nucleus accumbens and ventral midbrain. It also altered the serotonergic system in the ventral midbrain. These results suggest that the mesolimbic dopaminergic system is a primary target of repeated ATR exposure in female rats. It should be mentioned that the estrous stage in rats at the time of sacrifice varied enough so that we can discard possible hormonal influences on neurotransmitter tissue quantification in the brain regions evaluated. The observed increase in dopamine levels in the nucleus accumbens and ventral midbrain could be attributed to various mechanisms, including potential alterations in the enzymes responsible for dopamine degradation, such as monoamine oxidase (MAO) and aldehyde dehydrogenases, which could be influenced by ATR exposure. Previous studies have reported similar enzyme activity modifications due to pesticide exposure (Aziz and Knowles, 1973; Nag and Nandy, 2001; Goldstein et al., 2015).

Another possible explanation could be alterations in TH, such as increased phosphorylation. The regulation of tyrosine hydroxylase includes the inhibition by product and several transduction pathways linked to the phosphorylation by multiple kinases at four different serine residues (8, 19, 31, and 40) and dephosphorylation by two phosphatases. It has been reported that TH protein levels primarily control the long-term regulation of TH activity. In contrast, the acute regulation of TH activity occurs via phosphorylation of serine (Ser) residues (Daubner et al., 2011; Gordon et al., 2011). In this respect, Dunkley et al. (2004) reported that phosphorylation at serine 40 markedly increases TH activity *in vitro*, *in situ*, and *in vivo* (Dunkley et al., 2004). It is important to note that the acute injection of ATR (100 mg ATR/kg) increases the phosphorylation of TH at serine 19 and 40 (Rodriguez et al., 2007). However, in this study, the TH levels remained unaltered in the striatum or nucleus accumbens. Therefore, more studies are necessary to unveil the effects of ATR exposure on the regulation of this enzyme.

The alterations in locomotor activity observed in the present study could be associated with the modifications in dopamine and serotonin and their metabolites DOPAC and 5-HIAA in the nucleus accumbens and/or the ventral midbrain. These brain regions are part of the basal ganglia, which are involved in the control of motor behavior through DA, GABA, and glutamate regulation (Wu et al., 1993; Liu et al., 1998; Bolam et al., 2000; Salgado and Kaplitt, 2015). It is important to mention that in a similar protocol of exposure to ATR performed in male rats the hypoactivity was observed but the main alteration in monoamines was the decrease of DA, DOPAC, HVA and 5-HIAA in the striatum 5 days after ending ATR treatment (Rodriguez et al., 2013) and the decrease in binding to D1-DA receptor also in the striatum 2 months after ending ATR treatment (Marquez-Ramos et al., 2017). These data suggest the vulnerability of the mesolimbic dopaminergic system in female rats and comparison to the male rats where the nigrostriatal dopaminergic system appear to be more sensitive.

Supporting this finding is the decrease in dopamine levels in the striatum but not in the nucleus accumbens reported in male Sprague-Dawley rats chronically exposed to 10 mg ATR/kg BW for 1 year; these neurochemical changes were accompanied by impaired motor coordination, hyperactivity, and deficits in spatial memory (Bardullas et al., 2011; 2013). Interestingly, we recently reported that female Sprague-Dawley rats treated with 1 or 10 mg ATR/kg during 14 months showed vertical hypoactivity only in the first month of ATR exposure; nevertheless, the dopamine and its metabolites or TH protein levels in the striatum or nucleus accumbens were similar among ATR treated groups and the control group, suggesting that female rats could present greater sensitivity to the neurotoxic effects of ATR in the early stages of development (Sánchez-Yépez et al., 2024). All these studies suggest that repeated or chronic ATR exposure causes behavioral and neurochemical effects that are sex-dependent.

Regarding the increased levels of serotonin and 5-HIAA in the ventral midbrain, few studies have reported alterations in this neurotransmitter system due to ATR exposure. In this respect, our group previously reported a decrease in the striatal levels of 5-HIAA without alterations in the nucleus accumbens or ventral midbrain in male rats using a protocol like the one presented in this study (Rodríguez et al., 2013). A study using adult male C57BL/6 mice reported that exposure to ≥125 mg ATR/kg by oral gavage for 10 days increased the levels of the 5-HIAA in the striatum and prefrontal cortex (Lin et al., 2013). Another study on male Wistar rats treated with 50 mg ATR/kg by gavage from postnatal days 23–51 reported decreased serotonin-positive endocrine cells in the jejunum (Rajkovic et al., 2011). In zebrafish embryos exposed to 0.3 or 3 μg ATR/L throughout embryogenesis, significantly decreased 5-HIAA and serotonin turnover in adult female brain tissue but not in adult males; also, genes of the serotonergic pathway were downregulated at 9 months post-fertilization (Wirbisky et al., 2015).

Although exposure to ATR causes hypoactivity in both sexes, the results of our neurochemical analyses show that the affected dopaminergic pathways are different in male *versus* female rats. That means ATR exposure modified the mesolimbic pathway in female rats, while the nigrostriatal pathway was unaffected in this study. Several anatomic and physiologic factors can influence the different vulnerabilities associated with sex on the dopaminergic system. Several studies have reported that in adult female rodents, the acute treatment with estradiol increases dopamine release (Becker, 1990), upregulates dopamine D2 DA receptors binding (Bazzett and Becker, 1994) and DA transporter (DAT) activity (Calipari et al., 2017), and enhances behavioral responses to DA agonists (Cummings et al., 2014), for review see Yoest et al. (2018). Also, Wissman et al. (2011), Wissman et al. (2012) have reported in rats a higher distal dendritic spine density on medium spiny neurons in the nucleus accumbens in females in comparison to males, a rostrocaudal gradient in spine synapse density in females but not males in the caudal nucleus accumbens core and the higher percentage of spines with large spine heads in females in the rostral core in comparison to male rats.

Additionally, the excitability of the medium spiny neurons in the nucleus accumbens core depends on the estrous cycle in female rats (Alonso-Caraballo and Ferrario, 2019). In addition, alpha and beta-estradiol receptors are mainly abundant in VTA and nucleus accumbens in female rats (Almey et al., 2015). Estradiol regulates dopamine release and clearance in female rats (Becker, 1990). Considering that ATR may also influence the estrogen system in the dopaminergic system (Eldridge et al., 2008), and the anatomical and physiological characteristics just described, can help explain the differences between sexes presented by rats repeatedly or chronically treated with ATR.

The lack of alterations in the tissue levels of GABA, glutamine, and glutamate in the striatum, nucleus accumbens, prefrontal cortex, and ventral midbrain of female rats agrees with a previous study of our group, in which no alterations in the levels of these amino acids were found in these brain regions of male rats after the acute administration of 100 mg ATR/kg (Rodríguez et al., 2017). In contrast, another study reported that chronic ATR exposure to 1 or 10 mg ATR/kg during 13 or 14 months produces alterations in the extracellular levels of glutamate and tissue levels of GABA, glutamine, and glutamate on the striatum, nucleus accumbens, prefrontal cortex, and ventral midbrain among other brain regions (Chavez-Pichardo et al., 2020), in addition to the overexpression of genes related to the metabolism of these neurotransmitters in the same brain regions (Reyes-Bravo et al., 2022). This comparison among studies regarding the effects of ATR exposure on GABAergic and glutamatergic systems indicates that evaluating more extended periods of ATR exposure is necessary, that aging rats are more susceptible to ATR exposure, and the essential requirement for evaluating the effects of more prolonged exposure to ATR in aging female rats.

This groundbreaking study is the first to delve into the effects of repeated exposure to the herbicide ATR on motor activity and the dopaminergic/serotonergic/GABAergic/glutamatergic systems in adult female rats. The exploration of sexual dimorphism in neural vulnerability to pesticides, particularly in the context of early and adult pesticide exposures, remains unexplored (Gómez-Gimenez et al., 2017; Gómez-Gimenez et al., 2018; Shaffo et al., 2018; Tarazona et al., 2022; Berroug et al., 2024).

It is important to stress that this study while providing valuable insights, is just the beginning. Our present and previous findings unequivocally demonstrate that the herbicide ATR induces hypoactivity in both female and male rats, albeit through different neural pathways. These results underscore the critical need for further research to unravel the mechanisms of toxicity exerted by ATR, a task that requires the collective efforts of our scientific community.

## Data Availability

The raw data supporting the conclusions of this article will be made available by the authors, without undue reservation.
